# Effects of land-use change and disturbance on the fine root biomass, dynamics, morphology, and related C and N fluxes to the soil of forest ecosystems at different elevations at Mt. Kilimanjaro (Tanzania)

**DOI:** 10.1007/s00442-023-05353-6

**Published:** 2023-03-22

**Authors:** Natalia Sierra Cornejo, Joscha N. Becker, Andreas Hemp, Dietrich Hertel

**Affiliations:** 1 Plant Ecology and Ecosystems Research, Albrecht-Von-Haller Institute for Plant Sciences, University of Göttingen, Göttingen, Germany; 2 Department of Botany, Ecology and Plant Physiology, University of La Laguna, La Laguna, Spain; 3 Institute of Soil Science, CEN Center for Earth System Research and Sustainability, University of Hamburg, Hamburg, Germany; 4 Department of Plant Physiology, Bayreuth University, Bayreuth, Germany

**Keywords:** Carbon cycle, East Africa, Fine root system, Homegarden, Root litter nutrient flux

## Abstract

Tropical forests are threatened by anthropogenic activities such as conversion into agricultural land, logging and fires. Land-use change and disturbance affect ecosystems not only aboveground, but also belowground including the ecosystems' carbon and nitrogen cycle. We studied the impact of different types of land-use change (intensive and traditional agroforestry, logging) and disturbance by fire on fine root biomass, dynamics, morphology, and related C and N fluxes to the soil via fine root litter across different ecosystems at different elevational zones at Mt. Kilimanjaro (Tanzania). We found a decrease in fine root biomass (80–90%), production (50%), and C and N fluxes to the soil via fine root litter (60–80%) at all elevation zones. The traditional agroforestry 'Chagga homegardens' (lower montane zone) showed enhanced fine root turnover rates, higher values of acquisitive root morphological traits, but similar stand fine root production, C and N fluxes compared to the natural forest. The decrease of C and N fluxes with forest disturbance was particularly strong at the upper montane zone (60 and 80% decrease, respectively), where several patches of *Podocarpus* forest had been disturbed by fire in the previous years. We conclude that changes on species composition, stand structure and land management practices resulting from land-use change and disturbance have a strong impact on the fine root system, modifying fine root biomass, production and the C and N supply to the soil from fine root litter, which strongly affects the ecosystems' C and N cycle in those East African tropical forest ecosystems.

**Supplementary Information:**

The online version contains supplementary material available at 10.1007/s00442-023-05353-6.

## Introduction

The 60–85% of the world’s forests is subject to different kinds of anthropogenic use (IPCC 2019). Conversion of forests by human activities plays a major role for climate change as this is the second largest driver of greenhouse gas emissions (25% of the global emissions) (IPCC 2019). The increase of population and higher resource demands, together with the response to economic opportunities, depending on institutional factors, are among the main drivers of land-use change (Lambin et al. 2001). In addition, ecosystem disturbance can be intensified by climate change, e.g., through more aggressive fires caused by drier conditions (e.g., Hemp 2005). Among the terrestrial biomes, tropical forests are the most intensively used by humans, with 70% of their surface being fragmented or degraded by different activities, from intensive permanent agriculture to logging and attendant fire (Meiyappan and Jain 2012; Lewis et al. 2015; Mercer 2015). In particular, the total area of forest cover losses in Africa for the period 2000–2005 accounted for 11.5 Mio ha (Hansen et al. 2010) with the rates of forest loss in sub-Saharan Africa being among the fastest in the world (Fisher 2010).

Land-use change has strong effects on biodiversity, plant community composition and stand structure characteristics, as well as on climate regulation, physical properties of ecosystems and biogeochemical cycles (Foley et al. 2005; Canadell et al. 2007; Vitousek et al. 2008; Ensslin et al. 2015; Gerschlauer et al. 2016; Peters et al. 2019; Albrecht et al. 2021). Influential factors of the carbon (C) and nitrogen (N) cycle such as C storage and C allocation to plant compartments are consequently affected. Studying the impact of land-use change on plant components and dynamics is crucial to understand its effects on ecosystem functioning and processes.

Fine roots are the organs responsible for nutrient and water uptake. At the same time, they show rapid turnover rates, release exudates, establish symbioses with mycorrhizal fungi, and their litter represent an important substrate for the soil fauna and microorganisms (Jackson et al. 1997; Gill and Jackson 2000; Jones et al. 2005; Bardgett et al. 2014). They also shape the soil, affecting soil stability, porosity, and bulk density (Bardgett et al. 2014).

Fine root functions are tightly linked to their morphological and chemical traits. For instance, nutrient uptake is related to the absorptive surface as well as to the length of the roots to reach nutrient patches, reflected in the specific root area (SRA) and specific root length (SRL); the ability to penetrate compact soils and the storage capacity are related to root diameter; the carbon and nutrient storage, as well as the defense against herbivory and drought to root tissue density (RTD); and the metabolic activity to root N content (RNC) (e.g., Comas et al. 2009; Eissenstat et al. 2000; Freschet and Roumet 2017). Besides, these traits influence fine root lifespan (i.e., the inverse of turnover), which is a determinant factor in fine root activity, young roots being more active than older ones (Eissenstat et al. 2000; McCormack and Guo 2014; Weemstra et al. 2016).

The alteration of species composition, stand structure and soil properties driven by land-use change and disturbance regime affects fine root biomass, dynamics and morphological traits at the ecosystem level (Leuschner et al. 2009; Rajab et al. 2016). In addition, land-use management of agricultural systems entails very often the use of different practices (e.g., fertilizers, tillage, removal of plant litter) modifying the C and N cycle of the previous natural ecosystems (Allison and Vitousek 2004; Balesdent et al. 2000; Beer et al. 1998; Gerschlauer et al. 2016) and thus, the fine root system.

Although fine root traits are mostly genotypically determined, they are highly plastic and reveal a large intra- and interspecific variability. This characteristic enables plants to respond to changes in soil nutrient availability, as well as to coexisting species with a wide range of strategies of C investment into the fine root system (Pregitzer et al. 1993; Hodge 2004; Chapman et al. 2012; Valverde-Barrantes et al. 2013; Bardgett et al. 2014), which are based on a cost–benefit approach (Eissenstat et al. 2000). An acquisitive strategy invests resources on a high metabolic activity, entailing high nutrient uptake rates and short lifespan, together with a high specific root length (biomass per length unit) to explore and exploit more soil volume (Eissenstat et al. 2000; Weemstra et al. 2016). On the other hand, a conservative strategy holds lower metabolic rates together with long lifespan and high root tissue density to protect fine roots, e.g., against herbivory.

Another important component of the C and nutrient cycling is plant litter, as it represents the major source of organic matter to the soil. Land-use conversion affects fluxes of plant litter through changes in quantity and quality of litter production (N content, C:N ratio, lignin content). Both characteristics influence the soil microbial community composition and activity (Wardle et al. 2004; De Deyn et al. 2008; Bardgett et al. 2014) and thus, the soil N availability and C stocks. Fluxes of C and N to the soil via fine root litter have not received as much attention as the ones from leaf litter, especially in tropical ecosystems (Matamala et al. 2003; Röderstein et al. 2005). The study of fine roots is crucial to understand the changes on the C and N cycle along the plant–soil interface in a context of land-use change.

Forest ecosystems at Mt. Kilimanjaro (Tanzania) play a key role in the regional climate regulation, the provision of water, firewood as well as fertile land for food cultivation, among other benefits for the local communities (Hemp 2005; Agrawala et al. 2003). Mt. Kilimanjaro harbors a wide range of natural and disturbed ecosystems distributed in vegetation belts across the elevation (Hemp 2006). In the foothills, 85% of the savanna was converted to maize, millet and bean fields during the period from 1968 to 1985 (Soini 2005; Hemp and Hemp 2018). Upwards, from the lower montane forest until the border of Kilimanjaro National Park, around 1800 m a.s.l., two agroforestry systems sustain the local population. The shade coffee plantations function as an intensive land-use system and the traditional 'Chagga homegardens' (different crop plants under remaining natural forest cover) represent a sustainable land-use model (Soini 2005; Hemp 2006). In the middle montane forest zone (until 2600 m a.s.l.), selective logging of the dominant species, *Ocotea usambarensis*, took place until the year 1984. These forests patches are still regenerating (Rutten et al. 2015). In the case of the upper montane zone, areas of the forest dominated by *Podocarpus latifolius* have been replaced by *Erica excelsa* forest due to uncontrolled anthropogenic fires (honey collectors and poachers) and intensified by the drier conditions associated with climate change (Hemp 2005).

The wide range of natural and anthropogenic ecosystems occurring at Mt. Kilimanjaro gives the opportunity to assess the impact of land-use conversion and disturbance on ecosystem processes, such as the C and N cycle, not only occurring in this important tropical African region, but also contributing to our knowledge on land-use change effects at similar East African regions. Studies on the effects of land-use change and disturbance on aboveground and soil C stocks have already been developed at Mt. Kilimanjaro (e.g., aboveground carbon stocks: Ensslin et al. (2015); organic and microbial carbon stocks: Pabst et al. (2016); litterfall dynamics: Becker et al. (2015)), but still there are no data available concerning effects on the fine root system. Therefore, we studied fine root biomass and dynamics, C and N fluxes to the soil via fine root mortality and fine root morphological and chemical traits at four elevation zones, each one covering a different and unique set of natural and disturbed ecosystems. The present study follows up a recent study that was restricted to the natural forest zones (Sierra Cornejo et al. 2020). As the combination of natural and disturbed ecosystems is unique and different for each elevation zone, we only focus on the effect of land-use change and disturbance at a given elevation belt and not on the general effect of elevation (i.e., temperature regime, etc.). We aim to answer the following question: what is the magnitude of the effects of land-use change and disturbance on (i) fine root biomass and dynamics, (ii) C and N fluxes to the soil via fine root mortality, (iii) fine root morphological and chemical traits, and (iv) fine root C stocks compared to other C pools. Our hypotheses, addressed to the different elevation belts, are:

(i) in the pre-montane zone, the conversion of savanna woodlands to maize fields results in a decrease of fine root biomass and dynamics due to the change of the species life form (annual herbaceous vs. woody perennial);
(ii) in the lower montane zone, the traditional 'homegardens' and the intensive 'coffee plantations' show a lower fine root biomass than the natural lower montane forest as a response to a change on their stand structure and woody biomass;
(iii) in the pre-montane and lower montane zone, aboveground biomass is the C pool that decreases most due to land-use change as a result of change on species composition and stand structure, leading also in a decrease of soil C stocks;
(iv) in the middle montane zone, *Ocotea* forests' fine root system and fluxes might have recovered after 30 years of the cease of logging activity following the same trend than the aboveground stand properties in both disturbed and undisturbed ecosystems;
(v) in the upper montane zone, the replacement of *Podocarpus* forest by *Erica* species due to fire disturbance leads to a decrease in fine root biomass and fine root dynamics due to the large difference in aboveground structure, resulting in lower C and N fluxes from root turnover to the soil.

## Methods

### Study area

**Fig. 1:**
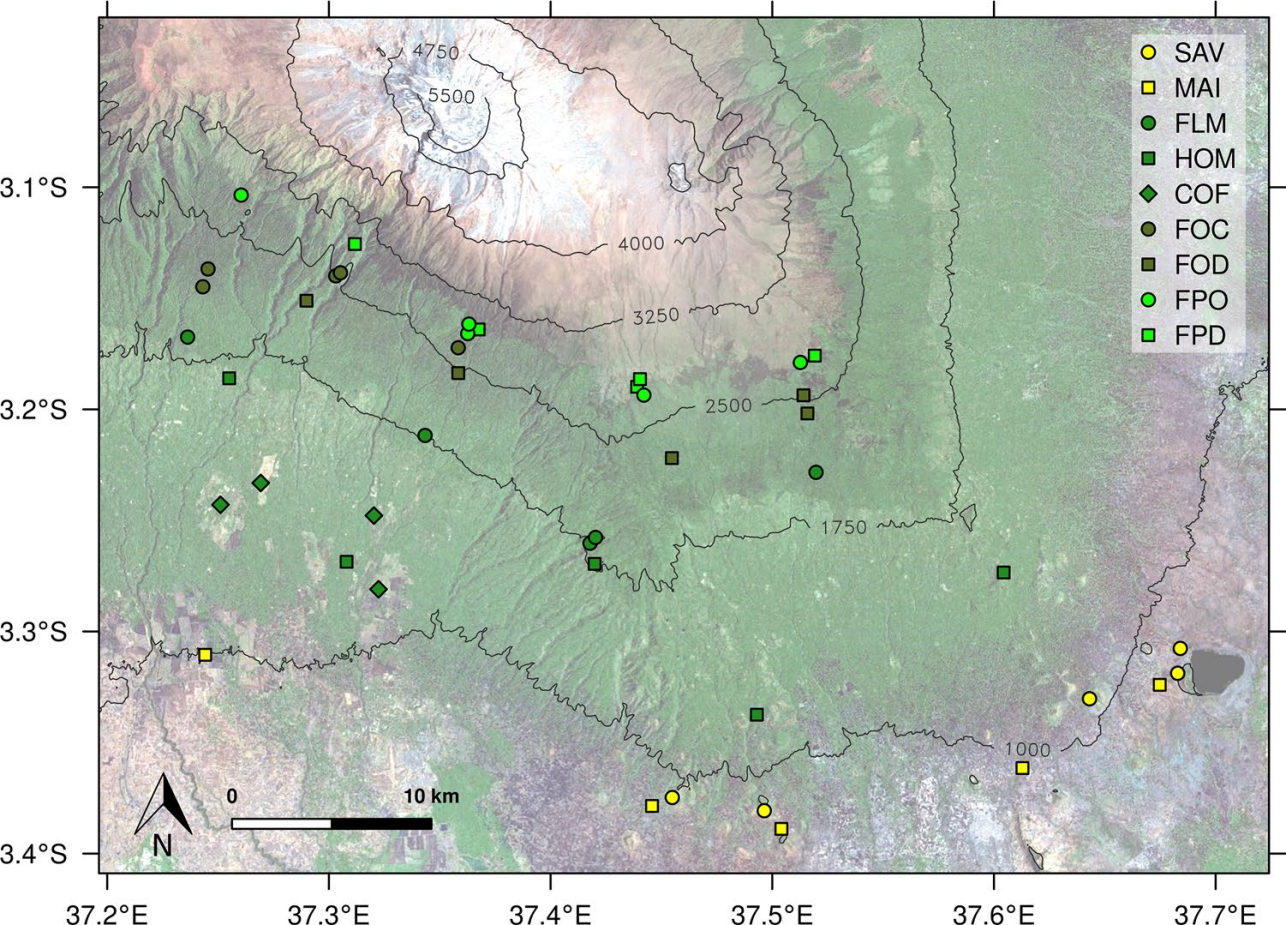
Bing Maps aerial image of the study area (downloaded via OpenStreetMap (2013)) and plots at the southern slopes of Mt. Kilimanjaro

This study is part of the KiLi project (DFG-FOR1246), an interdisciplinary framework with the aim of studying the effects of a climate and land-use gradient on biodiversity, biotic interactions and biogeochemical processes at the southern and south-eastern slopes of Mt. Kilimanjaro, in northern Tanzania (3°4´33´´S, 37°21´12´´E). Mount Kilimanjaro harbors a strong vertical zonation of vegetation belts (detailed description by Hemp 2006). We selected four of these elevation zones (pre-montane, low, middle, and upper montane forest types), containing natural (or semi-natural) ecosystems and areas of human-induced disturbance, hereafter called disturbed ecosystems (Table 1, Fig. 1). Each elevation zone harbors a unique set of natural and disturbed ecosystems. At the foothills of the mountain, in the pre-montane zone (800–1100 m a.s.l.), savanna woodlands, with *Acacia-Commiphora* trees, is the dominant semi-natural ecosystem. They are object to occasionally grass and wood cutting as well as irregular fires and have been increasingly converted into agricultural land (mainly maize fields) over the last decades (Soini 2005; Hemp and Hemp 2018). Forests in the lower montane zone (1200–2000 m a.s.l.), are dominated by *Macaranga kilimandscharica*, *Agauria salicifolia* and, to a lesser degree, *Ocotea usambarensis*, and are subject to low intensity logging of small stems for fire wood. Large forest areas were transformed into the traditional smallholder agroforestry system 'Chagga homegardens' (Hemp 2006) as well as intensively used shade coffee plantations. Chagga homegardens are a subsistence farming system and consist of a mixed cropping, mainly banana and coffee together with cultivated fruit trees (e.g., *Persea* *americana*) and shaded tolerant crops (e.g., taro, yams and beans) under remnant natural forest trees (e.g., *Albizia schimperiana*, *Grevillea robusta*). They are characterized by low inputs of organic fertilizers (dung and household waste) and pesticides and undergo regular manual ploughing. Coffee plantations (for commercial purposes) are intensive agroforestry systems consisting of coffee shrubs organized in rows (~ 2 m distance) under several shade trees (e.g., *Grevillea robusta, Albizia sp.*). Hose and aerial irrigation is used, and organic (urea) or/and inorganic (NPK) fertilizers as well as pesticides are applied several times per year. Litter from pruning is removed from the system. In the middle montane zone (2100–2800 m a.s.l.), the natural *Ocotea* forest is dominated by *Ocotea usambarensis*, *Ilex mitis*, *Xymalos monospora* and the tree fern *Cyathea manniana* (Hemp 2006). Due its high commercial value, *Ocotea usambarensis* has been a target for selective logging until the year 1984 (more than 30 years ago), when logging was banned (Agrawala et al. 2003), the disturbed forest areas still being under regeneration process (Rutten et al. 2015). The upper montane zone (2700–3100 m a.s.l.) hosts *Podocarpus latifolius* as the dominant tree species, together with *Hagenia abyssinica* and *Prunus africana*. In this zone, human-induced fires entailed the replacement of *Podocarpus latifolius* as the dominant species by *Erica excelsa*, which re-sprout from stumps (Hemp 2005).

**Table 1:** Characteristics of natural and disturbed ecosystems along the land-use gradient at Mt. Kilimanjaro. Mean ± SE (n = 5, Coffee plantations n = 4)

	Savanna (Sav)	Maize field (Mai)	Lower montane forest (Flm)	Homegarden (Hom)	Coffee plantation (Cof)	*Ocotea* forest (Foc)	*Ocotea* forest logged (Fod)	*Podocarpus* forest (Fpo)	*Podocarpus* forest disturbed (Fpd)
Land-use category	Natural, disturbed	Agricultural, intensive	Natural, disturbed	Agricultural, traditional	Agricultural, intensive	Natural	Disturbed	Natural	Disturbed
Elevation (m a.s.l.)	871–1130	860–1020	1620–2040	1150–1840	1120–1360	2120–2750	2220–2560	2720–2970	2770–3060
MAT (°C)	23.8 ± 0.5	23.8 ± 0.4	15.2 ± 0.4	18.6 ± 0.9	20.5 ± 0.9	11.6 ± 0.4	12 ± 0.5	9.4 ± 0.2	9.8 ± 0.4
MAP (mm)	820 ± 94	840 ± 49	2114 ± 139	1836 ± 233	1609 ± 136	2042 ± 192	1995 ± 156	1474 ± 109	1426 ± 102
SOC (g kg¯^1^)	26.5 ± 3.6	15.1 ± 2.35	137.9 ± 1.3	54.6 ± 10.7	36.6 ± 7.77	194.6 ± 12	188 ± 16	213 ± 11.2	199 ± 19.9
C:N soil	14.44 ± 0.90	11.57 ± 0.42	14.50 ± 0.96	12.19 ± 0.49	11.82 ± 0.54	18.80 ± 0.70	18.25 ± 1.21	18.80 ± 1.00	21.50 ± 1.18
pH (KCL)	5.38—7.27	4.56–6.34	4.23—5.30	4.45–5.93	4.01–5.64	3.49—4.25	4.32–4.39	3.83—5.35	4.00–4.85
VWC (%)	12,9	11,8	11,2	16,4	16,8	22,9	13,1	19,3	16,8
BD (g cm¯^3^)	1.01 ± 0.06	1.09 ± 0.03	0.45 ± 0.06	0.71 ± 0.08	0.89 ± 0.11	0.31 ± 0.02	0.33 ± 0.03	0.26 ± 0.02	0.32 ± 0.03
Texture (%) clay/silt/sand	27/34/39	36/44/20	45/41/14	44/39/17	46/39/15	48/41/10	47/41/11	44/47/9	42/46/13
AGB (Mg ha¯^1^)	10.0 ± 2.1 *	16.6 ± 2.5 *	360.1 ± 88.8	92.5 ± 17.2	58.0 ± 28.5	280.5 ± 48.8	356.6 ± 22.3	366.7 ± 3.5	…
Stem density (n ha¯^1^)	45 ± 12	…	388 ± 22	424 ± 23	226 ± 23	309 ± 20	598 ± 87	516 ± 76	1598 ± 378
Basal area (m^2^ ha¯^1^)	0.92 ± 0.24	…	49.50 ± 6.30	21.91 ± 3.97	11.84 ± 3.82	46.96 ± 5.26	62.58 ± 2.59	58.66 ± 3.62	40.54 ± 8.63
DBH (cm)	15.88 ± 0.94	…	35.43 ± 3.17	21.64 ± 1.96	24.78 ± 1.19	22.58 ± 1.19	23.47 ± 1.27	36.74 ± 3.11	18.87 ± 3.97
Mean tree height (m)	4.63 ± 0.26	…	17.68 ± 1.64	7.33 ± 0.70	9.14 ± 0.67	12.04 ± 0.89	13.96 ± 0.89	16.16 ± 1.25	8.94 ± 1.95
LAI (m¯^2^ m¯^2^)	0.77 ± 0.10	…	5.87 ± 0.30	2.64 ± 0.06	1.97 ± 0.30	5.32 ± 0.76	6.49 ± 0.43	5.30 ± 0.35	4.01 ± 0.38
LUI	0.13—0.35	0.53—0.79	0.08—0.17	0.45—0.63	0.95–1.00	0.00—0.01	0.07 -0.11	0.00—0.01	0.21—0.25

MAT: mean annual temperature, MAP: mean annual precipitation, SOC: soil organic carbon, VWC: volumetric water content, AGB: aboveground biomass, LAI: leaf area index, LUI: land-use index. AGB refers only to tree woody biomass except for (*) that refers total aboveground biomass. In homegardens and coffee plantations, stand structure variables refers to woody plant of all DBH sizes. In lower montane forest and *Ocotea* and *Podocarpus* natural and disturbed forest, stand structure variables refer to woody plants with DBH > 10 cm. MAT data from Appelhans et al. (2016); MAP, topographic and stand structure data from Hemp (unpublished data), Hemp (2006) and (Ensslin et al. 2015); soil data for topsoil (0–10 cm) from Becker (unpublished data), Pabst et al. (2015) and Gütlein et al. (2018); LAI data from Rutten et al. (2015); LUI data from Peters et al. (2019)

Our study design consists of five replicates each of natural (or semi-natural) and disturbed ecosystem types in their corresponding elevation zone (Table 1, Fig. 1). In total, 45 plots with 0.25 ha size were sampled. The tropical montane forest, *Ocotea* and *Podocarpus* disturbed forest plots are located inside Kilimanjaro National Park, while the savanna, maize, homegarden and coffee plantation plots are outside the protected area. The pre-montane zone (savanna and maize fields) was left out from the fine root morphological analyses due to the very different root morphological traits between maize (a grass) and savanna woody plants. Mean annual temperature along our elevational zones ranged from 25 °C in the savanna to 9 °C in the burned *Podocarpus* forest (Appelhans et al. 2016). Rainfall is characterized by a bimodal distribution with a long rainy season from March to May and a shorter one around November (Hemp 2006). Along the slope, mean annual precipitation exhibits a unimodal pattern with minimum values around 620 mm at the foothills and maximum values around 2600 mm at 2200 m a.s.l in the middle montane forest, followed by a decrease to 2050 mm in the upper montane zone (Hemp 2006; Appelhans et al. 2016). The soils on the Kilimanjaro Mountain all have roughly a similar age and developed from the same volcanic deposits (Dawson 1992). In the savanna, Vertisols have developed, while in the tropical montane forest, Andosols are predominant (Zech et al. 2014).

### Fine root biomass inventory

At each plot, 10 soil samples were taken at random locations down to 40 cm depth with a soil corer of 3.5 cm diameter and stored in plastic bags at 5 °C until processing. Nine of the 20 natural plots were the focus of a more intensive study; thus, 15 samples were collected in these plots instead. In the laboratory, samples were washed under running water over a sieve of 200 μm mesh size and all root fragments greater than 1 cm in length and ≤ 2 mm in diameter were selected. Under the stereomicroscope, fine roots were separated into biomass (living) and necromass (dead) fractions by means of the degree of root elasticity, the cohesion of cortex, periderm and stele, and the turgidity of the cortex (Leuschner et al. 2001). We further separated herb, grass and fern roots from woody tree (and shrub) roots by the lack of suberization. To compare the fine root bio- and necromass of the natural and disturbed ecosystems in the pre-montane zone (savanna and maize field plots) we joined together the data of woody and herbaceous roots. In the rest of the ecosystems, we only used woody fine roots data to develop the analyses. To estimate the root necromass of fragments lower than 1 cm in length, we followed the method introduced by Van Praag and others (1988) and modified by Hertel and Leuschner (2002). Six samples per plot were selected, and after extracting the larger root fragments (˃ 1 cm length) as described above, they were spread homogenously on a filter paper (730 cm^2^) subdivided into 36 squares. From six randomly selected squares, root fragments were extracted under the stereoscope, dry and weight. We then developed linear regression equations between the masses of the small root fragments and the larger dead fine root fractions. Finally, we applied these equations to the remaining samples that were not included in this more detailed analysis to obtain their small dead fine root fraction. When it was not possible to apply a regression equation, a mean ratio of small to large root fractions was used (ratio used in 25% of the plots). The root material was dried at 70 °C for 48 h, weighed and the fine root biomass and necromass were expressed in Mg ha^−1^ to 40 cm depth. Data for fine root biomass and necromass of one of the *Podocarpus* forest plots were missing due to logistic reasons and estimated using the mean value of the other plots from the same ecosystem type.

### Fine root morphological and chemical traits

Morphological traits of the living woody fine roots were investigated prior to drying. Each root sample was scanned using an EPSON perfection V700 scanner (EPSON America Inc.). Specific root length (m g^−1^), specific root area (cm^2^ g^−1^), mean root diameter (mm) and root tissue density (g cm^−3^) were calculated from data obtained through the scans using WinRhizo software (Regent Instruments Inc., Québec, Canada) and the respective fine root biomass data. In addition, we combined the information about root length and area per soil surface and estimated root length index (RLI) (root length per soil surface) and root area index (RAI) (root area per soil surface) per plot. We determined the C and N content of the living fine root fraction with a CN elemental analyzer (Vario EL III, Hanau, Germany). Three samples per plot were analyzed, with each sample consisting of two collected samples in the field and mixed. Data for C and N content of one of the *Podocarpus* forest plots were missing due to logistic reasons and estimated using the mean value of the other plots from the same ecosystem type.

### Fine root production and turnover

We used the ingrowth core approach to estimate annual woody fine root production (Majdi 1996). This method is very useful for studying differences in root production between sites when root growth is fast, as it happens in tropical forests (Vogt et al. 1998), as well as in studies comprising a large number of plots, as it is the case in our study. Due to our study design, it was not feasible to obtain enough replicates for each ecosystem using other methods, which are more time consuming in the field and in the laboratory (e.g., minirhizotrons, sequential coring). In September 2014 and February 2015 (dry season) we installed 10 ingrowth cores per plot at random locations in the topsoil down to a depth of 40 cm. After extraction of the soil with a corer of 3.5 cm in diameter, we removed all visible roots by hand and refilled the holes with the original root-free soil. We restored the original soil horizon sequence and soil bulk density as good as possible. Each location was precisely marked with a plastic tube with the same diameter as the soil core on top of the soil as well as with three thin plastic sticks. We resampled these locations after one year. Processing of the samples was done in the laboratory as described in the previous section and fine root production was calculated as fine root biomass growth into the cores related to the length of the period between the start of recolonization and harvest (Vogt et al. 1998). To determine the start of recolonization in the different studied ecosystems, we placed four additional ingrowth cores in every plot and resampled one of them every month (last core resampled after 4 months). Accordingly, recolonization started in the savanna, lower montane forest, homegarden and coffee plantation plots roughly two months after core installation; and in the middle and upper montane zones after three months. Fine root production values were extrapolated to one year and expressed as Mg ha^−1^ yr^−1^. In the case of the maize field plots, we assume that fine root production is equal to the total fine root mass (living plus dead), as maize is an annual crop. Fine root turnover was calculated at the plot level by dividing annual fine root production by mean standing fine root biomass (Gill and Jackson 2000). We assume a steady state between fine root mortality and productivity (Graefe et al. 2008). Due to missing ingrowth cores in the field, we estimated the fine root production of two coffee plots based on their fine root biomass and the mean ratio of fine root production to fine root biomass of the other coffee plots. Finally, we estimated for each ecosystem type the carbon and nitrogen fluxes to the soil via root mortality (equal to production) by multiplying the carbon and nitrogen concentrations in the fine root biomass by the corresponding fine root mortality rate, and expressed the flux in g m^−2^ yr^−1^.

### Coarse root, aboveground and soil C stocks

Coarse root C stocks were estimated indirectly from the aboveground C stocks reported in Ensslin et al. (2015) applying the equation by Cairns et al. (1997) (Eq. 1) for tropical forest sites.

1 $${\text{BGB}} = {\text{Exp}}\left[ {\left( { - 1.0587 + 0.8836\,{\text{ln}}({\text{AGB}}} \right)} \right].$$

BGB is the belowground biomass (in our case C stocks) in coarse roots and root stock in Mg ha^−1^ and AGB is the aboveground biomass (C stocks) in Mg ha^−1^.

Data on woody aboveground C stocks and soil C stocks down to 50 cm for the studied ecosystems was found in Ensslin et al. (2015) and Becker (unpublished data), respectively.

### Statistical analysis

All analyses (except the PCA) were conducted using R 3.4.0 software (R Development Core Team 2017). As each type of land-use change and disturbance is unique in each elevation zone, we do not compare ecosystems along the elevation. To determine differences on fine root biomass, necromass, productivity and root morphological traits among the natural and disturbed ecosystems, linear mixed effects models (LME) (function “lmer” in the “lmerTest” package (Kuznetsova et al. 2017) were applied in each elevational zone separately. We used all data points and designated “ecosystem” as fixed effect and “plot” as random effect. The Satterthwaite approximation of degrees of freedom was applied to correct for unbalanced sample numbers. In the case of fine root turnover, RLI, RAI, C and N fluxes and fine root N content, we used mean values per plot and applied ANOVA. For the lower montane zone, we used Tukey’s HSD post hoc adjustment for multiple comparisons (“glht” function in the package “multcomp” (Hothorn et al. 2008)) to detect differences between ecosystem types. Correlations among fine root biomass, necromass and dynamics and stand structure and chemical soil properties of the ecosystems at the lower montane zone was carried out with Pearson correlation (function “rcorr” from the package “Hmisc” (Harrell, 2022). A significance level of *p* < 0.05 was used throughout the analyses. When normality and homoscedasticity of model residuals were not found, log-transformation was applied to meet these criteria. In the case of samples with 0 values, we added one unit to all the data before log-transformation. Finally, we carried out a principal component analysis (PCA) to assess the interrelation of the fine root-related variables, stand structure and soil properties across the different natural and disturbed ecosystem types (only for the montane forest zones) using CANOCO software, version 5.02 (Biometris, Wageningen, the Netherlands).

## Results

### Fine root biomass, necromass, and dynamics of natural and semi-natural vs. disturbed ecosystems

Conversion of savanna woodland to maize fields in the pre-montane zone resulted on a three- and sixfold decrease of total fine root biomass (FRB) and necromass (FRN), respectively, and a decline to the half of fine root production (FRP) (Fig. 1). In the maize fields we did not find woody fine roots in our samples due to the low number of trees (0, 1 or 5) located in the edges of the plots. Moving upwards to the montane zones, in the lower montane belt, we found strong differences on all fine root studied variables with the transformation of the semi-natural lower montane forest to agroforestry systems (Fig. 1). Fine root biomass and necromass decreased around 70% and 75–90%, respectively, from lower montane forest to the agroforestry systems (Table S1). Lower montane forest and homegardens presented values of FRB: FRN close to 1, while fine root necromass in coffee plantations accounted for one third of fine root biomass (Table S1). Fine root production decreased the half from lower montane forest to coffee plantations, with no significant change in homegardens. However, homegardens exhibited the highest value for fine root turnover. At the middle montane zone, the differences on fine root turnover between *Ocotea* forest and selectively logged *Ocotea* forest were not significant (*P* = 0.11). The upper montane zone showed a decrease of threefold on fine root production from the natural *Podocarpus* forest to the burned *Podocarpus* forest, and a similar almost significant decrease on fine root turnover (*P* = 0.05).

### Root morphological and chemical traits of natural and semi-natural vs. disturbed ecosystems

**Fig. 2:**
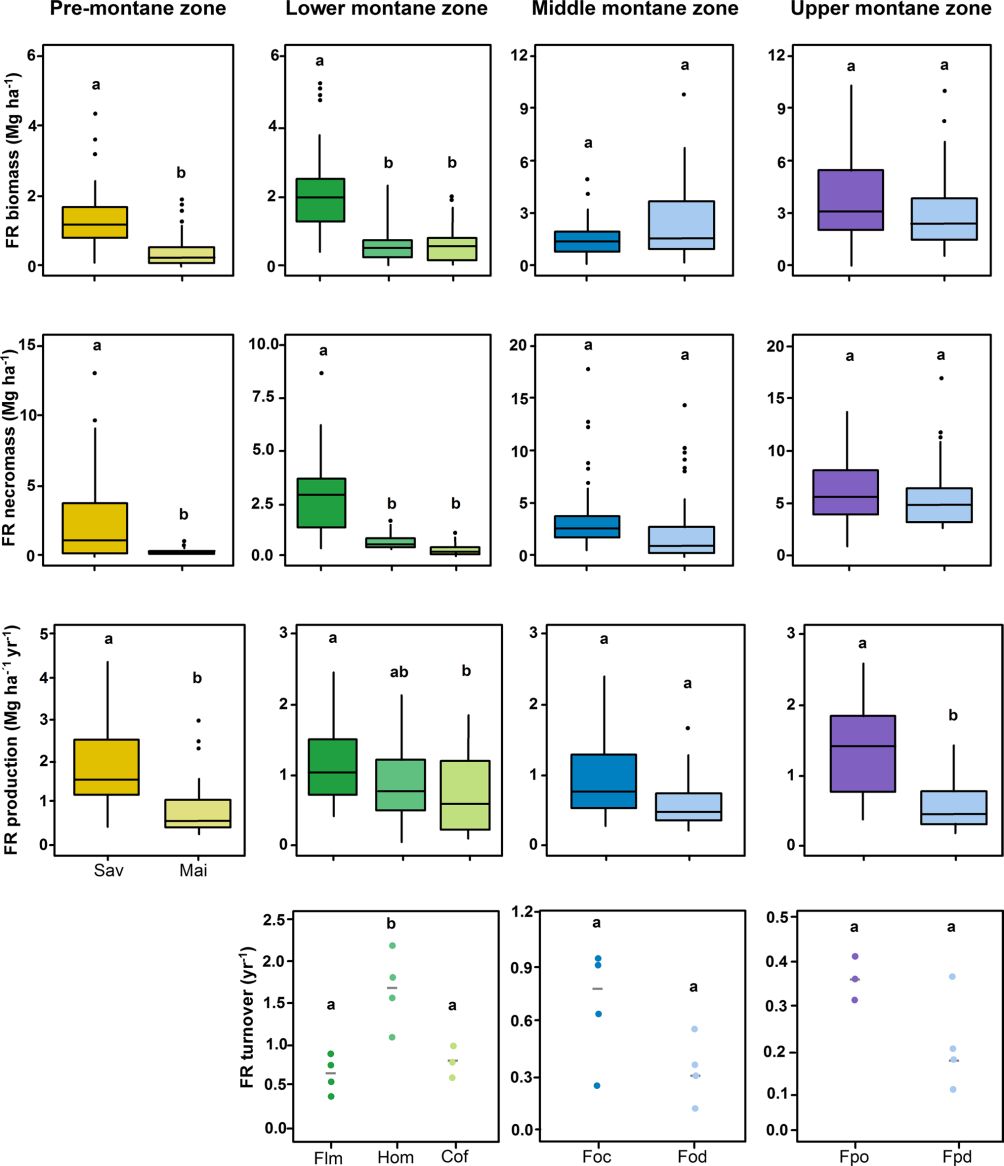
Fine root biomass and necromass, fine root production, and turnover in natural and disturbed ecosystems grouped by elevational zones at the southern slopes of Mt. Kilimanjaro. Different lower letters indicate significant differences between ecosystems following linear mixed effects models with Tukey HSD post hoc comparison (*P* < 0.05). Fine root turnover: dots are plot means and the gray line is the median. Notice that y-axes are in a different scale. Sav = savanna, Mai = maize field, Flm = lower montane forest, Hom = homegarden, Cof = coffee plantation, Foc = *Ocotea* forest, Fod = *Ocotea* forest logged, Fpo = *Podocarpus* forest, Fpd = *Podocarpus* forest disturbed

The traditional agroforestry homegardens showed a higher specific root length and specific root area, together with a lower mean root diameter and root tissue density compared to the lower montane forest (almost significant difference *P* = 0.05) (Fig. 2). In contrast, coffee plantations showed lower root N content than the lower montane forest and homegardens but did not differ from both ecosystems in the other fine root traits. In the *Ocotea* forest zone, disturbance did not affect fine root traits (Fig. 2). The burned *Podocarpus* forest presented a mixture of acquisitive and conservative fine root traits in the opposite direction than the one from its natural counterpart: a higher SRL and RTD, together with a lower mean root diameter and N content.

### Interrelationship among fine root traits, stand structure characteristics and soil properties

**Fig. 3:**
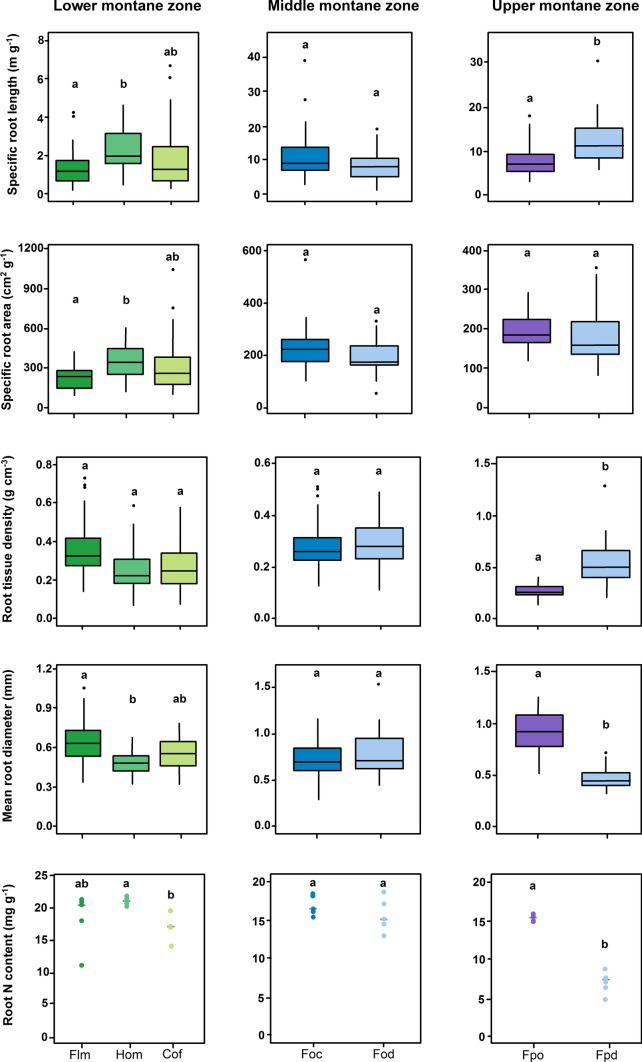
Fine root morphological and chemical traits in natural and disturbed ecosystems grouped by elevational zones at the southern slopes of Mt. Kilimanjaro. Different lower letters indicate significant differences between ecosystems following linear mixed effects models with Tukey HSD post hoc comparison (*P* < 0.05). Fine root N content: dots are plot means and the gray line is the median. Notice that y-axes are in a different scale. Flm = lower montane forest, Hom = homegarden, Cof = coffee plantation, Foc = *Ocotea* forest, Fod = *Ocotea* forest logged, Fpo = *Podocarpus* forest, Fpd = *Podocarpus* forest disturbed

**Fig. 4:**
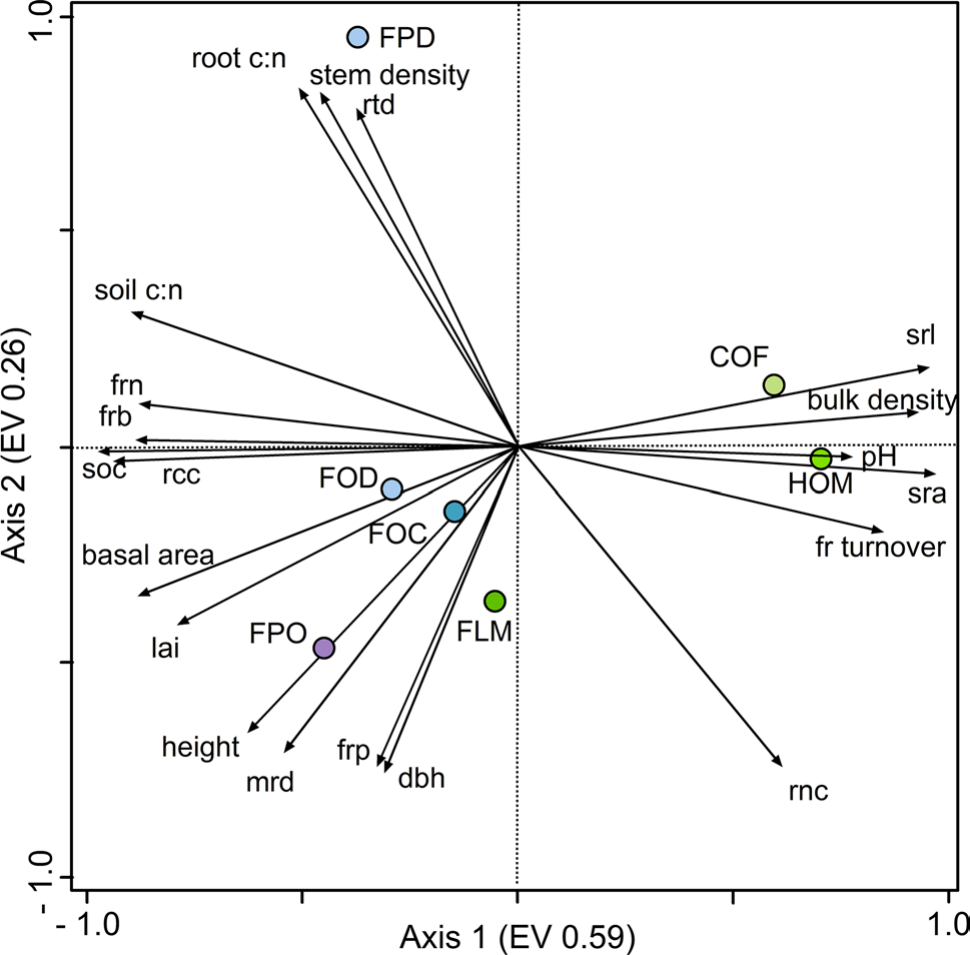
PCA plot showing the distribution of three natural forest ecosystems (FLM = lower montane forest, FOC = *Ocotea* forest, FPO = *Podocarpus* forest), two agroforestry ecosystems (HOM = homegarden, COF = coffee plantation) and two disturbed forest ecosystems (FOD = *Ocotea* logged, FPO = *Podocarpus* disturbed) at the southern slopes of Mt. Kilimanjaro, in relation to the PCA axes 1 and 2 (EV = eigenvalues of the axes) and their association with fine root-related variables, soil properties and stand structural characteristics. Vector length and angle are proportional to the direction and degree of their correlation with the plot ordination scores. Soil chemical data for topsoil (0–10 cm) from Becker (unpublished data), stand structure data from Hemp (unpublished data), LAI (Leaf Area Index) data from Rutten et al. (2015)

Ordination of the ecosystems (without the pre-montane zone) following principal component analysis (PCA) based on fine root-related variables, stand structural characteristics and soil properties established the differentiation of the ecosystems along the three elevation zones and distinguished natural from disturbed ecosystems (Fig. 3). Axis 1 mainly separated the elevational zones and axis 2 the land-use types. Most of the fine root-related variables, stand structural characteristics and soil properties were related to the first axis (eigenvalue = 0.59) (Table S2, Figs. 3,  4). This first axis markedly separated the agroforestry systems from the other ecosystems and associated them positively to SRA, SRL and bulk density. Soil organic carbon (SOC), soil C:N and FRB were among the variables most associated (negatively) with the first axis. The second axis (eigenvalue = 0.26) separated disturbed *Podocarpus* forest on one side and *Podocarpus* forest in the opposite side. Fine root production, mean root diameter, DBH and height were negatively interrelated to this axis in the direction of *Podocarpus* forest, while stem density, RTD and root C:N ratio where positively interrelated to the axis in the direction of burned *Podocarpus* forest. *Ocotea* forest and disturbed *Ocotea* forest were very close in respect to both axes, indicating strong similarities between them.

Pearson correlation analysis focused in the lower montane zone, showed a positive and significant correlation between fine root biomass and production with basal area, LAI and soil C:N, but fine root production did not hold any of these relations (Table 2).

**Table 2:** Correlations between fine root biomass, necromass, and dynamics and stand structure and chemical soil characteristics of the natural and disturbed ecosystems at the lower montane zone (FRB and FRN: n = 14; FRP and FRT: n = 12). Pearson correlation coefficients and P-values are shown. Statistically significant values (*P* < 0.05) are highlighted in bold

	Aboveground biomass		Basal area		Stem density		LAI		Soil C:N ratio		pH	
	*r*	*P*	*r*	*P*	*r*	*P*	*r*	*P*	*r*	*P*	*r*	*P*
FRB	0.44	0.11	**0.65**	**< 0.05**	0.19	0.52	**0.88**	**< 0.001**	**0.84**	**< 0.001**	**0.60**	**< 0.05**
FRN	0.26	0.37	**0.55**	**< 0.05**	0.36	0.21	**0.83**	**< 0.001**	**0.85**	**< 0.001**	**0.65**	**< 0.05**
FRP	0.55	0.06	**0.64**	**< 0.05**	0.50	0.09	**0.75**	**< 0.01**	0.50	0.10	0.35	0.32
FRT	0.33	0.30	0.26	0.41	0.53	0.07	0.43	0.16	0.54	0.07	0.49	0.15

Aboveground biomass data from Ensslin et al. (2015); basal area and stem density data from Hemp (unpublished data), LAI (Leaf Area Index) data from Rutten et al. (2015); soil chemical data for topsoil (0–10 cm) from Becker (unpublished data). FRB: fine root biomass; FRN: fine root necromass, FRP: fine root productions, FRT: fine root turnover

### Root area index and root length index of natural vs. disturbed forest ecosystems

The transformation of natural ecosystems did not affect the root length index (root length per soil surface) (RLI) nor the root area index (root area per surface) (RAI) in any of the elevation belts (Table 3). However, the change of lowland montane forest to coffee plantations resulted in a 2.5-fold decrease of both RLI and RAI, although the differences were not significant (*P* = 0.085 for RLI and 0.084 for RAI).

**Table 3:** Root length index (RLI) and root area index (RAI) of natural and disturbed ecosystems in three elevation zones along the southern slopes of Mt. Kilimanjaro. Lower case letters indicate significant differences on RLI or RAI among natural and disturbed ecosystems in the same elevational zone according to ANOVA and afterwards Tukey HSD post hoc comparison (*P* < 0.05). Mean ± SE (n = 5, coffee plantations and *Podocarpus* disturbed forest n = 4)

	Lower montane			Middle montane		Upper montane	
	Lower montane forest	Homegarden	Coffee plantation	*Ocotea* forest	*Ocotea* forest logged	*Podocarpus* forest	*Podocarpus* forest disturbed
RLI (m m¯^2^)	2763.0 ± 781.9 a	1344.1 ± 166.9 a	1096.9 ± 283.7 a	1540.8 ± 175.5 a	1964.0 ± 778.3 a	2803.7 ± 608.6 a	3641.7 ± 599.8 a
RAI (m^2^ m¯^2^)	4.5 ± 1.1 a	2.0 ± 0.3 a	1.8 ± 0.5 a	3.1 ± 0.4 a	4.3 ± 1.5 a	7.1 ± 1.0 a	5.3 ± 0.9 a

### *Carbon and nitrogen fluxes to the soil *via* fine root mortality*

Most of the land-use changes and ecosystem disturbances at Mt. Kilimanjaro resulted in a decrease of C and N fluxes to the soil via fine root mortality (Table 4). These fluxes refer to the C and N content in the fine roots that is deposited into the soil when fine roots die, without accounting for decomposition. At the lower montane zone, both fluxes decreased around a half from lower montane forest to coffee plantations, whereas homegardens maintained the fluxes and fine root N content values compared to the semi-natural forest. Both agroforestry systems (traditional homegardens and intensive coffee plantations) differ in their N fluxes and fine root N content, with lower values reported for the coffee plantations. Upward the mountain, in the middle elevation zone, no significant differences were found among selective logging and the natural *Ocotea* forest. The strongest decrease on N fluxes among all ecosystems were reported in the ecosystems in the upper montane zone, with an 81% lower value in the burned *Podocarpus* forest compared to its natural counterpart. The fine root N content also decreased a half, whereas the C:N ratio increased more than threefold in the disturbed *Podocarpus* forest. Neither C nor N fluxes were related to soil chemical properties as C:N or pH (Table S4).

**Table 4:** Estimated C and N fluxes to the soil through fine root mortality, N content and C:N ratio of fine roots in natural and disturbed ecosystems in four elevational zones in the southern slopes of Mt. Kilimanjaro. Lower case letters indicate significant differences between natural and disturbed ecosystems in the same elevational zone according to ANOVA and afterwards Tukey HSD post hoc comparison (*P* < 0.05). Mean ± SE (n = 4, *Ocotea* and *Podocarpus* disturbed forests n = 5, *Podocarpus* forest n = 3)

Elevation zone	Ecosystem	C flux (g m¯^2^ yr¯^1^)	N flux (g m¯^2^ yr¯^1^)	N (mg g¯^1^)	C:N ratio
Pre-montane	Savanna woodlands	72.8 ± 11.9 a	1.0 ± 0.1 a	6.1 ± 0.6 a	71.4 ± 8.1 a
	Maize fields	30.4 ± 14.8 b	0.9 ± 0.4 a	10.8 ± 0.9 b	37.2 ± 3.5 b
Lower montane	Lower montane forest	52.1 ± 5.2 a	2.3 ± 0.3 a	18.3 ± 1.9 ab	27.5 ± 3.7 a
	Homegarden	34.6 ± 2.5 ab	1.8 ± 0.2 a	21.3 ± 0.3 a	20.4 ± 0.4 a
	Coffee plantation	19.6 ± 5.4 b	0.8 ± 0.2 b	17.1 ± 0.9 b	27.8 ± 2.9 a
Middle montane	*Ocotea* forest	46.3 ± 13.4 a	1.6 ± 0.4 a	17.0 ± 0.6 a	28.9 ± 1.3 a
	*Ocotea* forest logged	28.4 ± 4.3 a	0.9 ± 0.1 a	15.9 ± 1.0 a	32.5 ± 1.8 a
Upper montane	*Podocarpus* forest	63.1 ± 3.0 a	2.1 ± 0.1 a	15.4 ± 0.2 a	31.9 ± 0.5 a
	*Podocarpus* forest disturbed	24.1 ± 3.6 b	0.4 ± 0.1 b	7.1 ± 0.6 b	72.5 ± 7.6 b

### Differences on aboveground and belowground C stocks with land-use change and disturbance

Besides the data of coarse and fine root C stocks obtained in this study, we also analyzed aboveground and soil C stocks data from Ensslin et al. (2015) and Becker (unpublished data) to get a general overview of the effects of land-use change and disturbance on the main C pools and on the total C stock of the studied ecosystems. Land-use change led to a strong decrease in the above- and belowground ecosystem C stocks, except for the middle montane zone and for the aboveground biomass in the pre-montane zone, where an increase was reported (Table 5). The highest decrease in C stock percentage was observed in the vegetation reservoirs (up to 80%), while soil C stocks experienced the smallest change for all types of land disturbance (up to 46%) (except between homegarden and coffee plantation where fine roots decreased a 9%). However, the magnitude of the change on soil C stocks is the largest, as this pool holds the highest soil organic carbon values. For instance, between lower montane forest and coffee plantations the difference in soil C stocks is of 120 Mg C ha^−1^ (46,6%), while a similar change on aboveground biomass (150 Mg C ha^−1^) implies the loss of 84% of this plant C pool (Table 5). Among all, the highest decrease in total reported C stocks (plant and soil) was from lower montane forest to coffee plantations (63%). At the upper montane forest belt, there are only available data for fine root and soil C stocks. At this elevation, a moderate decrease on fine roots C stocks happened (20% decrease) in comparison to the agricultural systems (70%), whereas no decline was reported in soil C stocks.

**Table 5:** Total and compartment (plant and soil) C stocks and the difference, in percentage, between natural and disturbed ecosystems at the different elevational zones at Mt. Kilimanjaro. Mean ± SE (n = 5, coffee plantations n = 4)

Elevation zone	Ecosystem	Carbon stocks (Mg ha¯^1^)				
		Aboveground C stocks	Coarse roots C stocks	Fine roots C stocks	Soil C stocks	Total C stocks
Pre-montane	Savanna woodlands	5.02 ± 1.00	1.02 ± 0.23	0.50 ± 0.05	109.65 ± 17.65	116.19 ± 16.52
	Maize fields	8.01 ± 1.18	0.20 ± 0.16	0.15 ± 0.02	70.38 ± 9.77	77.84 ± 10.69
Lower montane	Lower montane forest	173.58 ± 42.82	29.98 ± 6.55	0.90 ± 0.13	241.22 ± 45.74	445.67 ± 51.79
	Homegarden	44.60 ± 8.27	9.07 ± 1.50	0.24 ± 0.03	168.21 ± 24.15	222.13 ± 25.33
	Coffee plantation	27.94 ± 13.74	5.86 ± 2.53	0.26 ± 0.10	128.85 ± 19.63	162.91 ± 22.13
Middle montane	*Ocotea* forest	135.18 ± 23.54	24.17 ± 3.77	0.70 ± 0.11	278.96 ± 16.05	439.01 ± 22.27
	*Ocotea* forest logged	171.89 ± 10.73	30.06 ± 1.66	1.12 ± 0.34	300.90 ± 3.19	503.07 ± 67.70
Upper montane	*Podocarpus* forest	176.75 ± 1.86	30.84 ± 0.29	1.81 ± 0.43	295.55 ± 16.12	503.35 ± 17.09
	*Podocarpus* forest disturbed	…	…	1.44 ± 0.20	293.86 ± 2.66	…

Elevation zone	Ecosystem	Change in C stocks				
		Aboveground	Coarse roots	Fine roots	Soil	Total
Pre-montane	Savanna woodlands—Maize fields	+ 59.5%	− 80.4%	− 70.7%	− 35.8%	− 32.2%
Lower montane	Lower montane forest—Homegarden	− 74.3%	− 69.8%	− 72.9%	− 30.3%	− 50.2%
	Lower montane forest—Coffee plantation	− 83.9%	− 80.5%	− 70.0%	− 46.6%	− 63.4%
	Homegarden—Coffee plantation	− 37.4%	− 35.4%	− 9.2%	− 23.4%	− 26.7%
Middle montane	*Ocotea* forest—*Ocotea* forest logged	− 27.2%	+ 24.4%	+ 60.5%	+ 7.9%	+ 14.6%
Upper montane	*Podocarpus* forest—*Podocarpus* forest disturbed	…	…	− 20.4%	− 0.6%	…

( +) indicates an increase on C stocks with land-use change or ecosystem disturbance while (-) indicates a decrease. Aboveground C stocks data from (Ensslin et al. 2015); soil C stocks data (0–50 cm) from Becker (unpublished data)

## Discussion

### Effects of land-use change and disturbance on the fine root biomass, dynamics, and morphological traits of different tropical ecosystems

#### Pre-montane savanna woodland zone: impact of intensive agriculture practice

The conversion of savanna woodlands to maize fields represents a high impact on the fine root C stocks as it entails a 70% decrease of the fine root biomass. Our fine root biomass values for savanna were at the lower end of the range of values reported for other tropical savannas and dry forests (0.4–11.86 Mg ha^−1^) (Roy and Singh 1994; Chen et al. 2003; February and Higgins 2010; Moore et al. 2018). Differences might be due to the lower mean annual precipitation, the lower number of trees at our savannas and to its semi-natural condition, as they are subject to logging and burning pressure that has been intensifying during the last decades (Agrawala et al. 2003; Hemp and Hemp 2018). The values found for maize fields are similar to values reported in south Senegal and in Morogoro region in Tanzania (0.32 and 0.30 Mg ha^−1^, respectively) (Jonsson et al. 1988; Manlay et al. 2002).

In addition to the decrease of fine root biomass in maize fields compared to savannas, some of the key functions of fine roots (e.g., nutrient uptake, growth and rhizodeposition) are lost during the major part of the year (290 days), as maize plants are removed during harvest (roots remained). The marked decline of fine root biomass might also negatively affect physical soil stabilization, as fine roots facilitate the binding of soil particles, as well as enhance soil porosity and protect against soil erosion (Bardgett et al. 2014; Freschet and Roumet 2017). The remaining fine roots after harvest contribute with C and nutrients to the soil. Therefore, at this stage, maize fields present unidirectional C and N fluxes from dead fine roots to the soil, probably more open N cycle and physical soil destabilization.

#### Lower montane forest zone: extensive-traditional versus intense agroforestry

The decrease of fine root biomass, necromass, and production from semi-natural forest to agroforestry systems has also been reported in other studies in tropical regions (Hertel et al. 2009; Hundera et al. 2013). Our range of values for the fine root biomass and dynamics in the agroforestry systems is lower compared to estimations for shade coffee plantations and cacao agroforests: FRB: 1.6–2.2 Mg ha^−1^, FRP: 1.5–4.5 Mg ha yr^−1^ (Hertel et al. 2009; Leuschner et al. 2009, 2013; Abou Rajab et al. 2015; Defrenet et al. 2016). These studies were carried out in systems with much higher number of stems and mean annual precipitation compared to our study sites.

Land-use conversion entails changes in plant community composition together with stand structural and chemical soil characteristics. Regarding the effects of stand structure, a lower aboveground biomass (AGB) and leaf area index (LAI) led to a decrease in stand fine root biomass and production, in line with results reported for coffee plantations (Defrenet et al. 2016). Fine root biomass also responds to soil fertility, indicated by low values of soil C:N. All these variables are positively associated with the first axis of the PCA (as we did not have data on AGB for all ecosystems we included basal area and height, which are determinant factors of AGB). The effects of land-use change and disturbance on the fine root system are also reflected in the positive correlation between fine root biomass and production with some of the stand structural characteristics and soil C:N (not significant for FRP) following Pearson correlation. Although fine root production was not correlated to soil C:N, the two order higher fine root production to aboveground biomass ratio (FRP:AGB) in the agroforestry systems (Table S1), which also hold higher soil fertility values (lower soil C:N) compared to the lower montane forest, might indicate a higher investment of carbon in the fine root system when there is more N available, as reported in other studies (Pregitzer et al. 1993; Nadelhoffer 2000).

Our results point to homegardens as highly dynamic systems with high turnover rates, and more pronounced acquisitive traits than the lower montane forest, which might be a result of the plant species composition together with the effects of management practices. Although root traits are highly plastic in the adaptation to their environment, they are also phylogenetically driven, with different species having their particular set of features and strategies (Valverde-Barrantes et al. 2013, 2015). For example, much higher turnover rates have been reported for banana trees (2, 6 and 17 yr^−1^ for cord, secondary, tertiary and root hairs (Araya 2005) than for coffee plantations (1–1.3 yr^−1^) and tropical mountain forest (~ 0.8 yr^−1^) (Gill and Jackson 2000; Defrenet et al. 2016). At the same time, *Musa sp.* (banana) is known for being a fast-growing species able to have very high SRL (150 m g^−1^) (Turner and Barkus 1981). Acquisitive traits are related to high turnover rates (inverse of lifespan) (Eissenstat et al. 2000; Weemstra et al. 2016) in line with our results. In fact, the PCA separated homegardens from the other ecosystems based on the SRA, SRL and fine root turnover.

Management practices, for instance the density of shade trees, leaf litter and tillage, might also have a strong influence on fine root dynamics and traits. In homegardens, the reported high turnover rates might be indirectly facilitated by the presence of trees and leaf litter; through an effect on microbial activity and, thus, on N availability (García-Palacios et al. 2013; Horwath 2015; Gerschlauer et al. 2016). Moreover, a rapid fine root turnover is advantageous when competing for resources, as young fine roots are considered to be more active in nutrient uptake than old ones (Eissenstat et al. 2000). Although *Albizia* sp.*,* a characteristic species in homegardens, has been found to compete with coffee lateral roots, complementary niche use between species in similar agroforestry systems has also been reported (Dossa et al. 2008; van Asten et al. 2011; Defrenet et al. 2016). Acquisitive morphological traits are also beneficial in the homegardens root competition environment as they facilitate soil exploration (high SRL) and provide more area for a possible symbiosis with microorganisms (high SLA).

Fine root turnover in homegardens might also be triggered by tillage, as it enhances fine root mortality (Schroth 1998) and the consequent replacement of dead roots. The lower FRB:FRN ratio and high turnover rates found in homegardens compared to coffee plantations agree well with this idea. Tillage also destabilizes soil organic matter (SOM) facilitating N mineralization and improving the root system (Balesdent et al. 2000; Blomme 2002; Chen et al. 2009). High SRL might be advantageous after this practice as it enables fine roots to recover faster after disturbance, as it has been shown for arbuscular mycorrhizal trees (Eissenstat et al. 2015).

On the other hand, coffee plantations hold a less dynamic fine root system. Apart from the difference in species composition, both agroforestry systems are subject to different management practices. Coffee plantations present lower number of shade trees (also *Albizia* and *Grevillea sp.*), removal of pruned parts and addition of fertilizers (NPK). The lower leaf litter values and removal of pruned parts result on lower C substrate for microorganisms, which have to rely on dead fine roots and other C sources (e.g., root exudates, dead soil fauna and microorganisms, SOM) leading to a low gross N turnover (Gerschlauer et al. 2016).

The fine root length and surface per land area (RLI and RAI) are not affected by land-use conversion, as fine root morphological traits (SRL and SRA) compensate the low fine root biomass. LAI follows the same trend with land-use intensity as RAI does, in line with a land-use disturbance gradient in Indonesia (Leuschner et al. 2009).

#### Middle montane forests zone: effects of selective forest logging

Effects on the fine root system (biomass, necromass, morphological, and chemical traits) are not detectable 30 years after the end of the logging activity in *Ocotea* forest. Although fine root dynamics decrease almost a half from natural to disturbed ecosystems, this difference is not significant (turnover *p* = 0.11; production *p* = 0.18). These results indicate an almost complete recovery of the fine root system. In a study on fine root biomass regeneration of primary and secondary tropical forest, Hertel et al. (2007) suggested that the fine root system was recovered after 1–2 years since disturbance and did not find differences among fine root biomass of primary and secondary forest pulling data from different studies together. Fine root biomass recovery depends on stand age (Cavelier et al. 1996), which agrees well with our data as forest disturbance happened more than 30 years ago. However, the higher density of late successional species in the disturbed ecosystems (Rutten et al. 2015) can be noticed in the tendency of the fine root system to hold a higher fine root biomass, lower fine root dynamics and more conservative morphological and chemical traits, characteristic of slow growing species (Weemstra et al. 2016).

#### Upper montane forest zone: regenerated forest after burning

Fires at the upper montane zone of Mt. Kilimanjaro have led to a change of the plant species community, with *Erica excelsa* becoming the dominant species instead of *Podocarpus latifolius* (Hemp 2005), with consequences for the fine root system. The major effect was observed on fine root dynamics, as fine root production and turnover was c. 50% lower in the disturbed ecosystem. Although we assume that the disturbed forest presents a lower aboveground biomass due to the dominance of *Erica excelsa*, fine root biomass did not change. This fact indicates the high investment of carbon on the fine root system characteristic of *Erica* sp. together with the high SRL and SRA (Sierra Cornejo et al. 2020). The decline of fine root N content might indicate lower mineralization rates in the disturbed ecosystem (Hobbie et al. 2006). Both, *Podocarpus* and *Erica* sp. have adaptations to N shortage conditions. The distinctive fine root traits of the plant communities where they are the dominant species follow a contrasting mixture of conservative and acquisitive strategies. *Erica* sp. present high RTD, lifespan and low N content (conservative) and on the other hand, high SRL and fine root diameter (acquisitive), whereas patterns are the opposite in plant community dominated by *Podocarpus latifolius* (Sierra Cornejo et al. 2020). A high N content indicates high litter quality (Silver and Miya 2001), which entails easier decomposition by the microbial community. Both species present mycorrhiza symbiosis: arbuscular in the case of *Podocarpus* sp. and ericoid in *Erica* sp. (Khan 1967; Cairney and Meharg 2003). The opposite strategies of these species affect the C and N cycles through the nutrient uptake capacity, the fine root system size, and the quantity and quality of root litter. There might be a slow-down of the C and N cycle with the replacement of *Podocarpus latifolius* forest by *Erica excelsa* following events of fire, as already indicated by the lower fine root production, turnover, litter quality (N content) and the lower soil C:N values in the disturbed ecosystem.

As an overview, the factors that determine fine root biomass and dynamics differ among the type of ecosystems and disturbance. Change on species composition is crucial in all cases, particularly in the disturbed *Podocarpus* forest. Stand structure, soil fertility and management practices play a key role in agroforestry ecosystems, all of them being helping to explain the alteration of the fine root system with land-use change.

### Impact of land-use change and disturbance on ecosystem plant and soil C stocks

To assess the consequences of land-use change and disturbance on the main C stocks of natural (or semi-natural) ecosystems at Mt. Kilimanjaro, we used existing data from studies on woody aboveground C stocks (Ensslin et al. 2015) and soil C stocks (down to 50 cm) (Becker, unpublished data) carried out in the framework of the same project in Kilimanjaro region. Land-use conversion to agricultural and agroforestry systems has a strong impact on all C stocks, whereas the selectively logged *Ocotea* forest seems to be recovered after 30 years since disturbance and disturbed *Podocarpus* forest only shows, for the moment, a low impact on its fine roots and soil C stocks. The most affected studied component was plant C stocks, due to the change on stand structure and species composition (Ensslin et al. 2015). Although the percentage of soil C stocks decline is smaller compared to the C loss in the other components, soils contain the highest amounts of sequestered C among the studied C pools. Thus, the reduction of the soil C stock implies a large C loss. For instance, in the lower montane zone soil C reduction equals the C loss occurred in all the other plant components together. Studies have found that the decrease in plant litter and soil microbial biomass with land-use conversion, both characteristic of agricultural ecosystems, drives soil C stocks reduction (Pabst et al. 2015; Post and Kwon 2000). A detailed explanation about the effects of land-use change on the aboveground and soil C stocks at Mt. Kilimanjaro can be found in Ensslin et al. (2015) and Pabst et al. (2015) respectively.

The biggest difference on C stocks is displayed between lower montane forest and coffee plantations, reflecting the impact of intensive production systems. Management practices in agriculture and agroforestry systems are a crucial factor for soil C sequestration potential, as tillage, addition of fertilizers, removal of litter and release of Cu as fungicide affect the microbial activity and mineralization of SOM, as well as the fine root dynamics (Oikeh et al. 1999; Gale and Cambardella 2000; Tian et al. 2010; Pabst et al. 2015). The small decrease in fine root C stocks in the upper montane belts is due to the large fine root system of *Erica* sp. which dominates the disturbed forest. Changes on soil C stocks at this elevation zone might be seen in future years, as C fluxes from leaf litter will be extremely reduced (*Erica* sp. have a low litter fall (Sierra Cornejo et al. 2021) and the fine roots of *Erica* sp. have a long lifespan and low quality (Sierra Cornejo et al. 2021), which may reduce C supply to the microbial community and decomposition.

Decrease of C stocks with conversion to maize fields in the pre-montane zone will probably be extended across a larger area as a result of the predicted increase of the African population until the year 2050 (United nations 2013). But land-use change will also depend on the economic opportunities offered to the population by the institutions (Lambin et al. 2001). In addition, climate modulates the effects of land-use change on ecosystem functions, being arid ecosystems less resistant to alterations in a climate change context (Peters et al. 2019). In the case of homegardens, this ecosystem is endangered by the intensification of its production (there is no space for expansion), crop diversification and substitution for grasslands (Soini 2005; Maghimbi 2007). In coffee plantations, the yield production is decreasing as a consequence of higher temperatures, lower precipitations and management practices (Kumburu 2012; Craparo et al. 2015). Coffee cooperatives incentivize the use of shade trees, which might increase C stocks. Further studies in homegardens and coffee plantations are necessary to assess the effects of these new challenging conditions on ecosystem C stocks. Regarding the tropical montane forest, its protection under the Kilimanjaro National Park is crucial for the maintenance of ecosystem processes and interactions as well as for the population settlements in the entire region. It entails essential functions as regulation of watershed and climate and provides resources for the local communities, as firewood and non-timber products (Agrawala et al. 2003; Hemp 2005). The control of fires at the upper montane zone is a key factor to avoid changes on the water regime and C sequestration capacity.

### Effects of land-use change and disturbance on the C and N fluxes to the soil via fine root turnover

Land-use conversion and disturbance decreased soil C and N inputs via fine root litter (i.e., fine root mortality) compared to the natural ecosystems in almost all of the different elevational zones on Mt. Kilimanjaro. This was most pronounced in the upper montane zone. Similar effects were found in a study on the conversion of tropical rain forest to shaded cacao plantations and to different intensities of timber extraction in Indonesia (Hertel et al. 2009). This decrease entails a lower contribution to soil C stock and lower substrate amount for the decomposer community. It should be noted that we have assumed that there is no N retranslocation before root senescence (Ostertag and Hobbie 1999; Gill and Jackson 2000; Carrera et al. 2008). However, in evergreen forests, 13% of N is estimated to be retranslocated (Brant and Chen 2015), so our N fluxes might be slightly overestimated.

Although the fluxes from fine root litter to the soil are generally lower compared to leaf litter in the studied tropical ecosystems (Table S4) and in similar studies in the tropics (Hertel et al. 2009), the byproducts of root decomposition might play a key role in soil C storage (Rasse et al. 2005; Clemmensen et al. 2013). At maize fields, even when leaf litter is not removed, fine roots are an important C supply to the soil microbial community (Clapp et al. 2000; Kramer and Gleixner 2006). In addition, specific land-use practices and vegetation characteristics can increase the relative importance of belowground C inputs. At maize fields and disturbed *Podocarpus* forest, the strong decrease of leaf litter due to its removal in the first case and to the low quantity of litter fall in the second entails a large reduction of the plant contribution to soil carbon stocks. Thus, the microbial community must rely on fine roots, exudates, dead fauna and SOM among others as substrate. Moreover, the decline of the plant substrate amount (leaf and fine root litter) might entail microorganisms to decompose SOM (Kramer and Gleixner 2006), which leads to the destabilization of this important carbon pool. Besides, decomposition of plant litter is strongly influenced by its quality, which affects soil microbial community composition and activity (Makkonen et al. 2012; See et al. 2019; Wardle et al. 2004). At disturbed *Podocarpus* forest, the lower amount of available substrate, its lower quality and higher soil C:N compared to natural *Podocarpus* forest are other indicators of the possible slowing down of decomposition with forest disturbance, with consequences on soil C stocks and N availability.

Savannas and homegardens hold higher C and N fluxes to the soil via fine root litter mortality than maize and coffee plantations. This difference among land-use change is added to the high potential of these ecosystems for soil C sequestration, as they have lower decomposition rates and lower labile C decomposition compared to maize and coffee plantations, respectively (Mganga and Kuzyakov 2014; Becker and Kuzyakov 2018).

Specifically, our study highlights homegardens as a dynamic ecosystem, with high fine root turnover rates and high litter quality, high substrate availability and microbial efficiency (Pabst et al. 2015). Despite the changes of species composition and stand structure, homegardens keep some of the properties from the lower montane forest. Their multilayer vegetation structure and crop diversity contribute to maintain high biodiversity, leaf litter production and high gross N turnover rates, (Hemp 2006; Becker et al. 2015; Gerschlauer et al. 2016). To these processes, we add the maintenance of the C and N fluxes from fine root mortality to the soil and the root litter quality, which plays an important role on the plant-soil interface pathway of the C and N cycle. In summary, C and N fluxes are mainly driven by changes on species composition and management practices.

## Conclusions

The increase of land-use transformation worldwide, especially in tropical ecosystems, urges assessment of its effects on ecosystems components and fluxes. Fine roots, being typically underrepresented in this kind of study, play a crucial role on ecosystem processes like the C and N cycles. Our investigation on a wide range of land-use types at different elevation zones at Mt. Kilimanjaro (Tanzania) showed a strong decrease of the fine root biomass, production and turnover with anthropogenically land-use change or disturbance in almost all studied forest ecosystems. A decrease in the C and N fluxes to the soil via fine root death was also observed, especially in the disturbed *Podocarpus* forest (upper montane zone). Results from traditional 'Chagga' homegardens pointed to particularly high fine root turnover rates. The variation of plant species composition, stand structure and, to a lesser extent, management practices such as leaf litter removal, tillage and the use of fertilizers or cow manure are among the responsible factors of the changes on ecosystem processes. In addition, land-use change entails a shift of the fine root litter quality, which is considered to drive the microbial community composition and activity, playing, indirectly, a major role in C and N cycles.

Change on plant species composition leads to a different matrix of fine root morphological traits, as we observe in the acquisitive fine root traits at homegardens, driven by the community species composition, and the mixture of traits at disturbed *Podocarpus* forest, driven by the dominance of *Erica excelsa*. We highlight the agroforestry system 'Chagga homegarden' as it maintains properties and processes from the semi-natural forest, such as high fine root turnover rates, similar C and N fluxes from fine root mortality to the soil and fine root litter quality, while providing resources to the local communities. Our study results suggest that further studies are urgently needed for a better understanding of belowground plant strategies and the implications of land-use change for tropical ecosystem processes related to the C and N cycles at different elevational locations.

## Supplementary Information

Below is the link to the electronic supplementary material.

Supplementary file1 (PDF 83 KB)

## Data Availability

The datasets analyzed during the current study are available from the GoettingenResearchOnline: https://doi.org/10.25625/ERCJFJ
