# Supplementary material for: Effects of land-use change and disturbance on the fine root biomass, dynamics, morphology, and related C and N fluxes to the soil of forest ecosystems at different elevations at Mt. Kilimanjaro (Tanzania)
Source: Oecologia. 2023 Mar 22;201(4):1089–107. doi: 10.1007/s00442-023-05353-6 (PMC10113319; doi:10.1007/s00442-023-05353-6)
Supplement: Supplementary file 1 — Supplementary file1 (PDF 83 KB) [file 442_2023_5353_MOESM1_ESM.pdf]

Supplementary information

Table S1. Means of fine root biomass (FRB), necromass (FRN), productivity (FRP), and turnover, and ratios of FRB to aboveground biomass, FRP to aboveground biomass, and FRB to FRN in the different natural, semi-natural and disturbed ecosystems on Mt. Kilimanjaro. Mean ± SE (fine root biomass and necromass: n=5, coffee plantations n=4; fine root production and turnover: savanna and maize fields, *Ocotea* and *Podocarpus* disturbed forest n=5; lower montane and *Ocotea* forest, coffee plantations and homegardens n=4,; *Podocarpus* forest n=3).

	Fine root biomass (Mg ha^{-1})	Fine root necromass (Mg ha^{-1})	Fine root production (Mg ha^{-1} yr^{-1})	Fine root turnover (yr^{-1})	FRB:AGB	FRP:AGB	FRB:FRN
Savanna woodlands	1.22 ± 0.11	2.51 ± 0.49	1.78 ± 0.28	0.74 ± 0.07	0.14 ± 0.04	0.22 ± 0.07	0.56 ± 0.11
Maize fields	0.39 ± 0.04	0.42 ± 0.04	0.80 ± 0.08	…	0.03 ± 0.01	…	0.94 ± 0.07
Lower montane forest	1.97 ± 0.27	2.53 ± 0.71	1.13 ± 0.11	0.65 ± 0.11	0.008 ± 0.002	0.003 ± 0.001	1.06 ± 0.26
Homegarden	0.57 ± 0.07	0.61 ± 0.05	0.82 ± 0.06	1.67 ± 0.23	0.007 ± 0.001	0.011 ± 0.002	0.94 ± 0.12
Coffee plantation	0.62 ± 0.24	0.22 ± 0.03	0.46 ± 0.13	0.80 ± 0.08	0.018 ± 0.009	0.012 ± 0.005	2.75 ± 0.76
Ocotea forest	1.46 ± 0.21	3.40 ± 1.04	0.95 ± 0.27	0.68 ± 0.19	0.007 ± 0.002	0.005 ± 0.002	0.54 ± 0.12
Ocotea logged	2.29 ± 0.72	2.65 ± 1.52	0.58 ± 0.08	0.33 ± 0.07	0.006 ± 0.002	0.002 ± 0.000	1.67 ± 0.55
Podocarpus forest	3.74 ± 0.32	6.51 ± 0.59	1.33 ± 0.05	0.36 ± 0.03	0.010 ± 0.001	0.004 ± 0.000	0.60 ± 0.11
Podocarpus forest disturbed	3.01 ± 0.41	5.63 ± 0.79	0.50 ± 0.07	0.19 ± 0.05	…	…	0.56 ± 0.08

Aboveground biomass data from Ensslin et al. (2015)

)

Table S2. Results of a Principal Components Analysis (PCA) on the differentiation of three natural ecosystems and four disturbed ecosystems with respect to fine root biomass, dynamics and traits, soil properties of the topsoil and stand structural characteristics. Given are the loadings of the selected variables along three explanatory axes. Numbers in brackets indicate the eigenvalues of the axes and numbers in bold mark the variables with closest correlation to the respective axis.

	**Axis 1 (EV 0.59)**	**Axis 2 (EV 0.26)**	**Axis 3 (EV 0.07)**
Soil properties			
SOC	**-0.97**	-0.02	0.09
Soil C:N ratio	**-0.91**	0.32	0.11
pH	**0.76**	-0.04	-0.54
Bulk density	**0.95**	0.08	-0.05
Stand structural variables			
Stem density	-0.46	**0.83**	-0.25
Basal area	**-0.88**	-0.35	0.11
Height	-0.63	**-0.67**	-0.15
DBH	-0.32	**-0.75**	-0.53
LAI	**-0.80**	-0.41	0.22
Fine root variables			
Fine root biomass	**-0.91**	0.01	-0.35
Fine root necromass	**-0.89**	0.10	-0.32
Fine root production	-0.31	**-0.75**	-0.44
Fine root turnover	**0.87**	-0.20	-0.07
Fine root traits			
Specific root length	**0.96**	0.18	-0.11
Specific root area	**0.98**	-0.06	-0.01
Mean root diameter	-0.55	**-0.72**	0.08
Root tissue density	-0.38	**0.79**	-0.24
Root carbon content	**-0.95**	-0.03	0.25
Root nitrogen content	**0.62**	-0.75	0.12
Root C:N ratio	-0.51	**0.84**	-0.18

Soil chemical data for mineral topsoil (0-10 cm) from Becker (unpublished data), stand structure data from Hemp (unpublished data), LAI (Leaf Area Index) data from Rutten et al. (2015).

Table S3. Correlations of carbon and nitrogen fluxes to the soil through fine root mortality and soil chemical properties in natural and disturbed forest plots (n=29). Pearsson correlation coefficients and *P*-values are shown.

		Soil C:N			pH	
		r	*P*		*r*	*P*
FR C Flux		0.18	0.36	-	0.35	0.13
FR N Flux	-	0.21	0.28	-	0.18	0.44

FR: Fine roots. Soil C:N data in the topsoil and pH from Becker (unpublished data)

Table S4. Carbon and nitrogen fluxes to the soil through leaf litter and its N content and C:N ratio in semi-natural and agroforestry systems in the lower montane zone at Mt. Kilimanjaro. Mean ± SE (n=5, Coffee plantations n=4).

Elevation zone	Ecosystem	C flux (g m^{-2} yr^{-1})	N flux (g m^{-2} yr^{-1})	N (m g^{-1})	C:N ratio
Leaf litter*	Lower montane forest	216.9 ± 0.7	4.9 ± 0.2	1.1 ± 0.1	44.9 ± 0.5
	Coffee plantation	223.1 ± 16.0	11.0 ± 0.8	2.4 ± 0.1	20.4 ± 0.6
	Homegarden	270.1 ± 124.7	16.03 ± 7.33	2.9 ± 0.1	17.0 ± 0.1

* Data from Becker et al. (2015)
